# Evidence of sp-d Exchange Interactions in CdSe Nanocrystals Doped with Mn, Fe and Co: Atomistic Tight-Binding Simulation

**DOI:** 10.3390/nano16020122

**Published:** 2026-01-16

**Authors:** Pruet Kalasuwan, Worasak Sukkabot

**Affiliations:** 1 Division of Physical Science, Faculty of Science, Prince of Songkla University, Hat Yai, Songkhla 90110, Thailand; pruet.kal@gmail.com; 2 Department of Physics, Faculty of Science, Ubon Ratchathani University, 85 Sathollmark Rd., Warinchamrab, Ubon Ratchathani 34190, Thailand

**Keywords:** tight-binding method, sp-d exchange interaction, Zeeman splitting, g-factor, nanocrystals

## Abstract

Exploiting the atomistic tight-binding theory with the sp-d exchange term, the electronic and magnetic characteristics of CdSe nanoparticles embedded with Mn, Fe and Co are determined as a function of external magnetic fields to realize the sp-d exchange interactions. The transition metal species and applied magnetic fields are powerful factors to manipulate the electronic and magnetic characteristics of doped CdSe nanoparticles. With growing applied fields, the energies of spin splitting, Zeeman splitting and magnetic polaron improve and are assumed to reach saturation at high fields. All g-factor values are boosted in the presence of the external field and then fade with increasing applied fields. The electron spin-splitting energies and electron g values are ordered as Fe:CdSe > Mn:CdSe > Co:CdSe. The single-particle gaps, hole spin-splitting energies, Zeeman splitting energies and hole g values follow the order Co:CdSe > Fe:CdSe > Mn:CdSe.

## 1. Introduction

The ability to intentionally incorporate magnetic ions into nanocrystals has unlocked exciting opportunities for building spintronic nanodevices [1,2,3,4]. The crucial knowledge of the physics of the magnetism involving the sp-d exchange interactions between host carriers and magnetic ion spins is primarily used to explain well-known diluted magnetic semiconductors [5]. Because of the sp-d exchange interactions, giant Zeeman splitting energies are demonstrated, and these lend them a diversity of astonishing magnetic, magneto-optical and magneto-transport characteristics [6,7,8]. Giant excitonic Zeeman splitting yields the giant Faraday rotation effect [9,10,11], the spin-polarized excitonic photoluminescence [12] and the spontaneous zero-field magnetization [13,14,15,16]. When integrating the underlying physics of the dilute magnetic semiconductor into the quantum confinement effect, the sp-d exchange interactions improve, and then their electronic, optical and magnetic characteristics are significantly influenced [17,18,19]. In the past decade, II-VI and III-V dilute magnetic semiconductors have been intensely investigated both from theoretical and experimental techniques for spintronic devices [20,21,22,23,24,25]. Among all, the CdSe nanocrystal has emerged as an efficient contender for applications including solar cells [26], spintronics [2], light-emitting diodes [27] and biomedical tags [28]. Numerous studies have endeavored to synthesize and characterize CdSe nanocrystals doped with several transition metals. Woo-Chul Kwak et al. [29] and Nor Aliya Hamizi et al. [30] proposed the inverse micelle technique to synthesize Mn doping in CdSe nanocrystals. Vitaly Proshchenko and Yuri Dahnovsky [31] studied the content dependence of magnetic order in CdSe nanocrystals doped with Mn using a more accurate ab initio CASSCF method. The ferromagnetic order was demonstrated at small distances between the Mn atoms, while the antiferromagnetic order was conversely realized at larger distances. Moreover, 3% Fe:CdSe and 3% Mn:CdSe nanostructures with the suppression of surface defects were synthesized by S. K. Shinde et al. [32]. These dopant nanostructures could be implemented for miscellaneous applications. Jasleen K. Bindra et al. [33] proved the ferrimagnetism in 4% Fe-doped CdSe nanocrystals. Sayantani Das et al. [34,35] synthesized pure and Fe-doped CdSe nanocrystals by the soft chemical route. Fe dopants in CdSe nanocrystals mainly affected the electronic and optical properties [34]. The antiferromagnetic order of the Fe-doped CdSe nanocrystals was confirmed [35]. Using the hydrothermal method, CdSe nanoparticles doped with Fe ions were successfully synthesized by Jaspal Singh and N. K. Verma [36]. The room-temperature ferromagnetic order and the saturation magnetization of Fe-doped CdSe nanocrystals increased with increasing Fe doping concentration. D. V. Sridevi et al. [37] presented a simple procedure for synthesis of Fe-doped CdSe nanocrystals using the precipitation technique with mercaptoethanol as the capping agent. Fe doping in CdSe nanoparticles delayed the recombination of electron and hole pairs. Amar Nath Yadav et al. [38] prepared isolated 0.5% Fe-doped CdSe quantum dots. The multiple magnetic transitions were revealed. Alina M. Schimpf et al. [39] used variable-temperature magnetic circular dichroism (MCD) spectroscopy to study the Zeeman splittings of the excitons in CdSe nanocrystals doped with Co and Mn at low dopant contents. The exchange interactions dominated at low temperatures, while the intrinsic Zeeman interactions were pronounced at room temperature. Jing Wang et al. [40] synthesized Co-doped CdSe nanocrystals by a co-precipitation method in homogeneous solutions. The electronic structure and magnetic properties of CdSe quantum dots doped with Co were experimentally studied by Joshua T. Wright et al. [41]. Superparamagnetism was suggested in Co-doped CdSe quantum dots. Jaspal Singh and N. K. Verma [42] synthesized CdSe nanoparticles doped with Co by a hydrothermal technique. The transition from ferromagnetism to antiferromagnetism was demonstrated with increasing concentrations. Jiwoong Yang et al. [43] reported the effective incorporation of Co ions into tremendously small-sized CdSe clusters. The smallest Co-doped CdSe cluster was formed by one or two Co ions substituted in the (CdSe)_13_ cluster.

In this work, we present the electronic and magnetic properties of CdSe nanocrystals manipulated by magnetically doping systems with Mn, Fe and Co to understand the mechanism of the sp-d exchange interactions and their possible applications. To address the dependence of the sp-d exchange interactions on the transition metal species, the resulting calculations are determined as a function of the exterior applied magnetic fields using the atomistic tight-binding calculations with the sp-d exchange term. In this manuscript, Section 2 portrays the Hamiltonian of the nanocrystals doped with different transition metals. The calculations in Section 3 are undertaken (i) to analyze the electronic properties of CdSe nanocrystals doped with several species of magnetic ions as a function of the applied fields and (ii) to use the energies of spin splitting to assess the nature of the sp-d exchange interactions and to investigate how the sp-d exchange terms are affected by magnetic ion species and external magnetic fields. Finally, the resulting calculations are summarized in Section 4.

## 2. Theoretical Details

The underlying physics to describe the electronic and magnetic behaviors of dilute magnetic semiconductor nanocrystals relies on the sp-d exchange interaction of doped magnetic ions with host carriers. For a theoretical description, the total Hamiltonian (H_T_) of CdSe nanocrystals doped with transition metals is built up from the contributions of the electronic host band structures and sp-d exchange interaction by [44]

(1) $$H_{T}=H_{\mathrm{host}}+H_{\mathrm{sp}-d}$$

where H_host_ is the host Hamiltonian of undoped CdSe nanocrystals responsible for their electronic properties. The last term H_sp-d_, the Kondo-like exchange Hamiltonian, is included to account for the sp-d exchange interaction. Each Hamiltonian will be described in the following.

We begin with H_host_. This term is first described by the crystal structure of the CdSe nanocrystals. The existing experimental data [32,33,35,36,41] reveals that CdSe nanocrystals adopt the wurtzite lattice structure. Using their atomic positions, the single-particle spectra are calculated via the nearest-neighbor sp^3^s^∗^ basis tight-binding model. From the second quantization description, the tight-binding Hamiltonian of the host is described by [45]

(2) $$H_{\mathrm{host}}=\sum_{i=1}^{N}\sum_{\alpha =1}^{m}\varepsilon_{i\alpha }c_{i\alpha }^{†}c_{i\alpha }+\sum_{i=1}^{N}\sum_{\alpha =1}^{m}\sum_{\alpha^{\prime }=1}^{m}\lambda_{{i\alpha \alpha }^{\prime }}c_{i\alpha }^{†}c_{i\alpha^{\prime }}+\sum_{i=1}^{N}\sum_{i^{\prime }=1}^{N}\sum_{\alpha =1}^{m}\sum_{\alpha^{\prime }=1}^{m}t_{i\alpha ,i^{\prime }\alpha^{\prime }}c_{i\alpha }^{†}c_{i^{\prime }\alpha^{\prime }}$$

Here, the summation goes over all atoms (N) and all atomic spin orbitals (m) on a given atom. $c_{i\alpha }^{†}$ and $c_{i\alpha }$ are represented as the creation and annihilation operators of the carrier with an α spin-orbital term on atomic site i, respectively. $\varepsilon_{i\alpha }$ and $t_{i\alpha ,i^{\prime }\alpha^{\prime }}$ are the on-site energies and hopping parameters between the nearest-neighbor orbitals. Attributed by the work of Chadi [46], the spin–orbit interaction is described by the $\lambda_{{i\alpha \alpha }^{\prime }}$ term, which is only contributed by the p orbitals. Using the CdSe tight-binding parameters [47], its bulk band gap is reproduced to agree with the experimental data [48,49]. To exclude non-physics phenomena such as non-radiative recombination and gap states, the energy shift of the dangling bond is applied on the nanocrystal surface [50]. The energy shift of 25 eV is applied to the dangling bond orbitals of all surface atoms. The surface effect is not considered in this work.

The last term is responsible for the sp-d exchange interaction describing the transition metal doping in the CdSe nanocrystals. For the demonstration, H_sp-d_ is expressed by

(3) $$H_{sp-d}=\sum_{R_{i}}J^{sp-d}(r-R_{i})S_{i}•\sigma$$

Here, S_i_ and σ are represented as the operators of the studied transition metal and host spin, respectively. J^sp-d^ terms are denoted as the sp-d exchange interactions. In addition, the summation goes over all transition metal ions (R_i_). J. K. Furdyna [44] theoretically simplified this H_sp-d_ term with the assitance of the mean-field theory and virtual crystal approximation. Therefore, the revised H_sp-d_ is given by

(4) $$H_{sp-d}=\sigma_{z}\langle S_{z}\rangle x\sum_{R}J^{sp-d}(r-R)$$

The summation goes over all cations (R). x and $\sigma_{z}$ are the content of the magnetic ions and the Pauli-spin vector in the z direction. Due to the simplification by J. K. Furdyna [44], the paramagnetic system is considered. The magnetic field is applied to the magnetically doped nanocrystals in the z direction. Then, the spin expectation term $\langle S_{z}\rangle$ is simplified as the thermal average [44,51]:

(5) $$\langle S_{z}\rangle =SB_{S}\left(\frac{g\mu_{B}SB}{k_{B}T_{\mathrm{eff}}}\right)$$

Here, the Brillouin function B_s_ is described by the effective temperature $T_{\mathrm{eff}}$, Bohr magneton value $\mu_{B}$ and Boltzmann constant $k_{B}$. S, g and B are the transition metal spin (5/2), g-factor value (2.0) and magnitude of the applied fields, respecively. In principle, the sp-d exchange term contains s-d and p-d exchange contributions. The s-d exchange term is attributed to the interaction between d orbitals of transition metal ions and s-like electrons of the host crystals. The s-d exchange term introduces the spin splitting of the cations with the s orbital. Then, the Hamiltonian used to describe the spin splitting of the s-like cation at the positions R is expressed as

(6) $$H_{s-d}=\pm \langle S_{z}\rangle xN_{0}\alpha$$

Here, the spin-up and -down states are described by + and − signs, respectively. N_0_ is symbolized as the number of unit cells in the unit volume. $\alpha =\langle s|J^{sp-d}|s\rangle$ are the s-like electron exchange integrals. The $N_{0}\alpha$ values of the studied transition metal doping in the CdSe semiconductor are listed in Table 1 [52]. The p-d exchange interaction theoretically describes the interaction between d orbitals of transition metal ions and p-like electrons of host nanocrystals and then produces the spin splitting of the cations possessing the p orbital at the position R. The Hamiltonian of the spin splitting by p-d exchange term becomes

(7) $$H_{p-d}=\pm \langle S_{z}\rangle xN_{0}\beta$$

Here, $\beta =\langle p_{i}|J^{sp-d}|p_{i}\rangle$ stands for the exchange integrals of p-like electrons with p_i_ orbitals oriented in the i axis, which represents x, y or z. The $N_{0}\beta$ values of the studied transition metal doping in the CdSe semiconductor are itemized in Table 1 [52]. After the total Hamiltonian matrix is generated, the single-particle spectra around the band gap are numerically attained from the PRIMME Version 2.0 software [53,54]. The resulting calculations are interpreted as qualitative or semi-quantitative trends. Using this model, the electronic and magnetic properties of the nanocrystals and hetero-nanocrystals magnetically doped with transition metals have been successfully studied [17,18,19,55].

## 3. Results and Discussion

**Figure 1:**
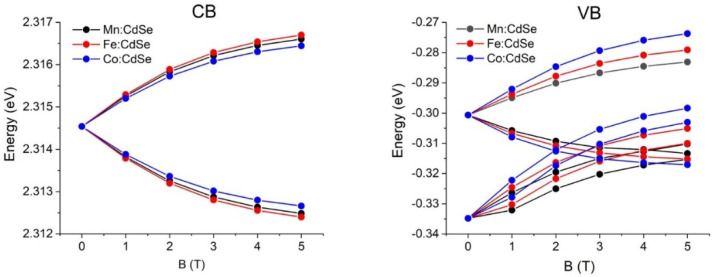
Single-particle spectra of Mn:, Fe: and Co:CdSe nanocrystals as a function of the external applied magnetic fields. CB and VB represent the conduction and valence bands, respectively.

**Figure 2:**
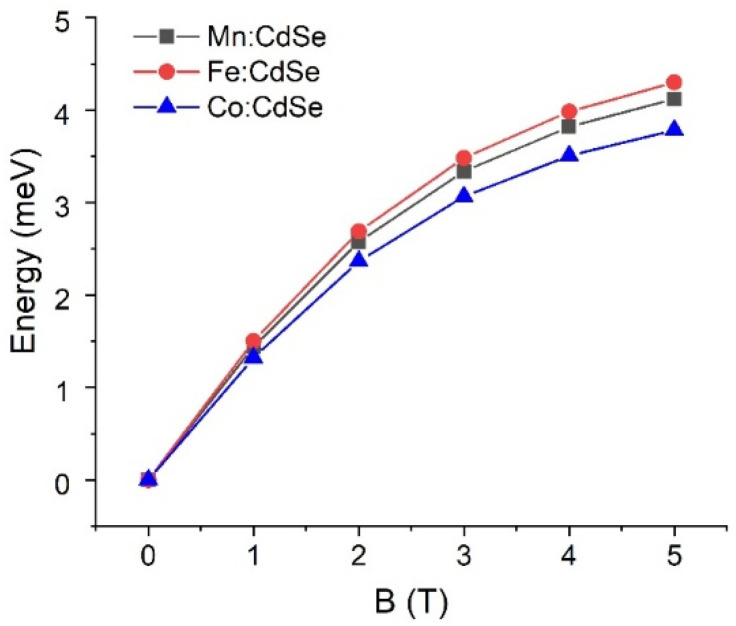
Electron spin-splitting energies of Mn:, Fe: and Co:CdSe nanocrystals as a function of the external applied magnetic fields.

**Figure 3:**
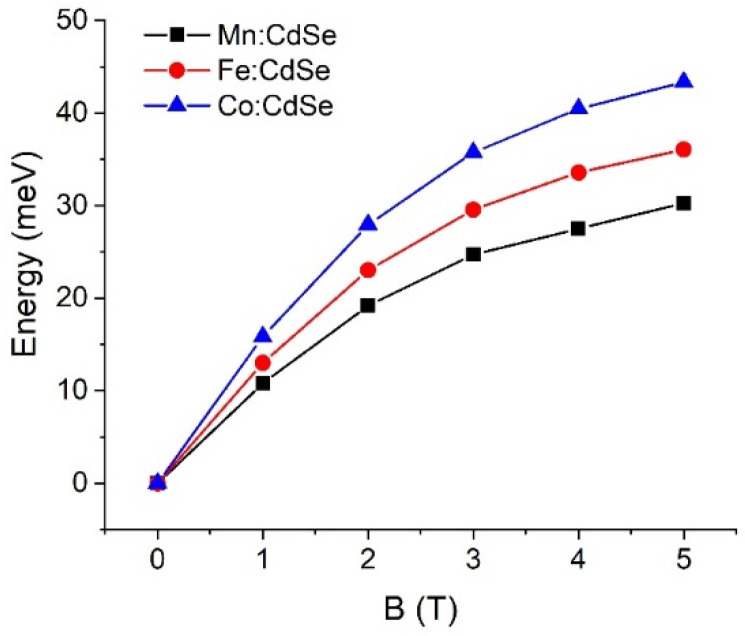
Hole spin-splitting energies of Mn:, Fe: and Co:CdSe nanocrystals as a function of the external applied magnetic fields.

The control of the magnetic exchange interactions by governing the species of the transition metals and the strengths of the magnetic fields is theoretically realized. Exploiting the atomistic tight-binding model with the additional sp-d exchange interaction of the host carrier with magnetic ion spins, we determine the electronic and magnetic characteristics of CdSe nanocrystals doped with Mn, Fe and Co as a function of the applied magnetic fields along the *z*-axis. The calculated crystallite diameter of the Mn:, Fe: and Co:CdSe spherical nanoparticles is fixed at 3.0 nm, which lies in the experimentally synthesized ranges [29,41,42]. The number of atoms inside the studied nanocrystal is 501. Here, Cd atoms are substituted by Mn, Fe and Co atoms with the doped concentration of 5.0%. Like the size of the nanocrystal, this doped concentration falls within the ranges of the experimental studies [36,37,40,41,42]. In addition, samples are operated at effective temperatures of 5 K. The first evidence to realize the effect of transition metal species doping in CdSe nanocrystals is the single-particle spectrum of the electrons and holes in Figure 1. For the demonstration, the numbers of the electron and hole states are 2 and 4 with the consideration of the spin degeneracy, respectively. The ground electron and hole states are s-like and heavy-hole-like characters, respectively. In the presence of the applied magnetic fields, the spin degenerate states of both electrons and holes are broken and then give rise to the splitting between spin-up and spin-down states because of the sp-d exchange term. As can be seen from the distribution of the energy spectrum, the transition metal species obviously make the contribution to the valance bands rather than to the conduction bands. These calculated results related to the spin splitting will be verified in the subsequent part. Interestingly, there is a crossing point occurring at the valence bands at the external magnetic fields above 2.0 T. However, there is no crossing point happening at the conduction bands over the entire field range. The crossing points of the valence band in the presence of the magnetic field have a physical effect on the hole character and the selection rules. When the external magnetic field is higher than 2.0 T, the characters of the excited hole states are changed, thus leading to the alteration of the selection rules. Next, the sp-d exchange interactions in electrons and holes are manipulated by the transition metal species and the strengths of the applied fields. The spin-splitting energies are the direct evidence to determine and verify this physics. Figure 2 and Figure 3 display the ground electron and hole spin-splitting energies, respectively. The spin-splitting energies of electron and hole states are responsible for the s-d and p-d exchange interactions. The results emphasize that the s-d and p-d exchange interactions can be manipulated by the transition metal species and external magnetic fields. With increasing magnetic fields, the s-d and p-d exchange interactions mainly improve and are expected to reach saturation at high fields. The hole spin-splitting energies are much higher than the electron spin-splitting energies because the p-d exchange integrals describing the interaction of the holes with magnetic ion spins are higher than the s-d exchange integrals representing the interaction of electrons with magnetic ion spins. The descending order of the electron spin splitting is Fe:CdSe > Mn:CdSe > Co:CdSe. As can be attributed by the p-d exchange interacting parameters, the calculations envisage that the hole spin splitting follows the order Co:CdSe > Fe:CdSe > Mn:CdSe. Used in this way, spin splitting is established as an efficient tool for probing and distinguishing magnetic exchange interactions in dilute magnetic nanocrystals.

**Figure 4:**
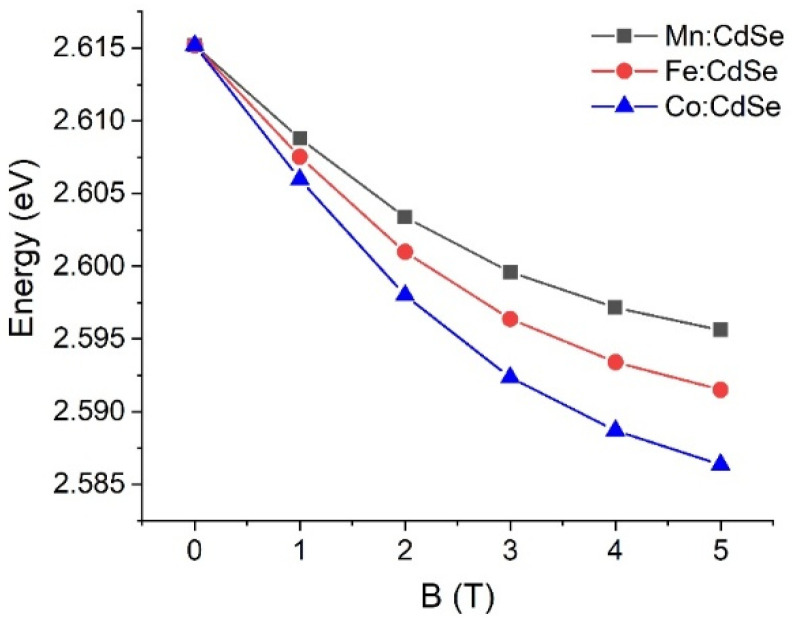
Single-particle gaps of Mn:, Fe: and Co:CdSe nanocrystals as a function of the external applied magnetic fields.

**Figure 5:**
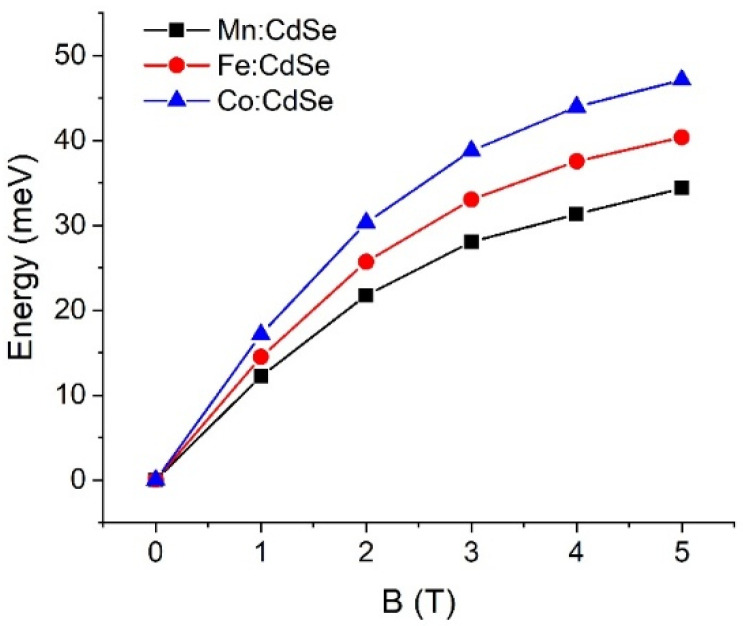
Zeeman splitting energies of Mn:, Fe: and Co:CdSe nanocrystals as a function of the external applied magnetic fields.

To realize the optoelectronic device’s performance, Figure 4 shows the calculated single-particle gaps of CdSe nanocrystals doped with Mn, Fe and Co as a function of the applied magnetic fields. As can be seen from the single-particle spectra in Figure 1, the single-particle gaps are narrow with increasing magnetic fields. The calculations highlight that the single-particle gaps follow the order Co:CdSe > Fe:CdSe > Mn:CdSe. These results are attributed to the sp-d exchange interactions and spin-splitting energies. In addition, the optical spectra of all studied nanocrystals are found to be slightly red-shifted with increasing fields. By changing the doped species and the strengths of the magnetic fields, luminescence within the visible spectrum (474.13 nm to 479.63 nm) is successfully demonstrated and verified for the realization of the spin optoelectronic device. The Zeeman splitting is one of the main factors used to determine the sp-d exchange interaction between the exciton spins and the transition metal ions in the presence of the exterior magnetic field. Here, the energies of Zeeman splitting are computed by the relation $E_{\sigma^{-}}-E_{\sigma^{+}}$, with $E_{\sigma^{-}}$ and $E_{\sigma^{+}}$ denoting the excitonic energies emitting $\sigma^{-}$ and $\sigma^{+}$ photon, respectively. The Zeeman splitting energies of CdSe nanocrystals doped with Mn, Fe and Co are calculated under several external magnetic fields in Figure 5. Analogous with the electron and hole spin splitting, the Zeeman splitting energies are progressively enhanced and estimated to be saturated at high fields. The augmentation of Zeeman splitting energies with increasing fields confirms the strong sp-d exchange interaction of these studied nanocrystals. The Zeeman splitting energies follow the order Co:CdSe > Fe:CdSe > Mn:CdSe. The results clearly demonstrate that the splitting of excitonic energies emitting $\sigma^{-}$ and $\sigma^{+}$ photons can be adjusted within simply accessible magnetic fields and transition metal species, thus finding relevance for upcoming spin-photonic information processing technologies. Alina M. Schimpf and Daniel R. Gamelin [39] reported the saturated Zeeman splitting of Cd_0.995_Mn_0.005_Se and Cd_0.995_Co_0.005_Se nanocrystals with 4 meV and 10 meV, respectively. According to the computational parameters (diameters, magnetic ion concentrations and magnetic fields), our calculations are not comparable with this experiment. If the magnetic ion concentrations, one of the computational parameters, are reduced to match with the experiment, the Zeeman splitting energies are close to the experimental data.

**Figure 6:**
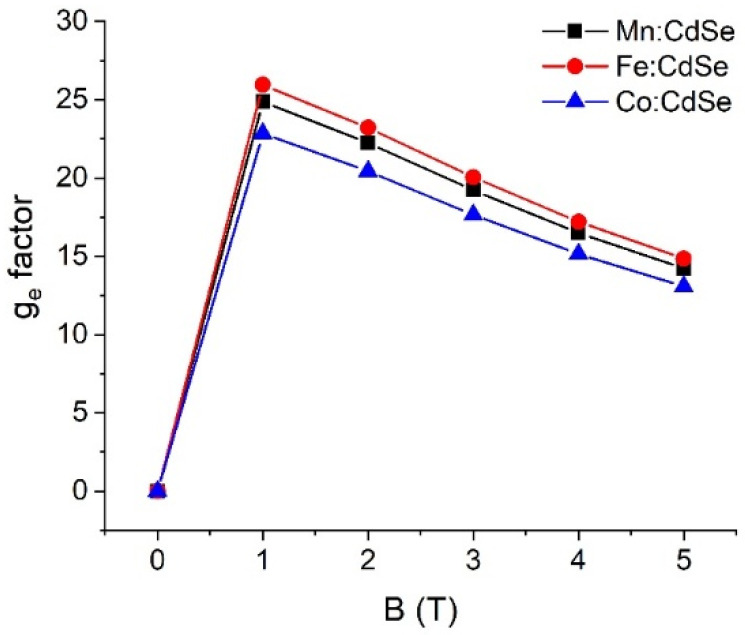
Electron g-factor values (g_e_) of Mn:, Fe: and Co:CdSe nanocrystals as a function of the external applied magnetic fields.

**Figure 7:**
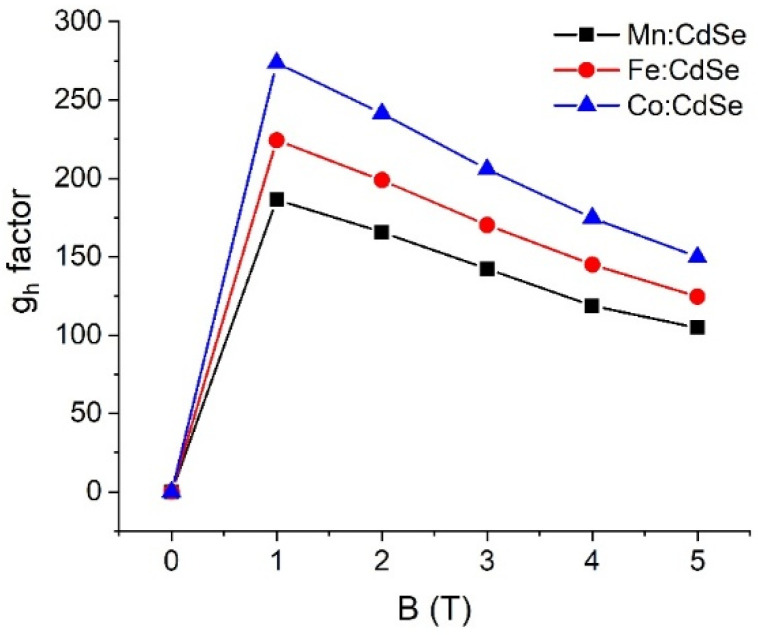
Hole g-factor values (g_h_) of Mn:, Fe: and Co:CdSe nanocrystals as a function of the external applied magnetic fields.

**Figure 8:**
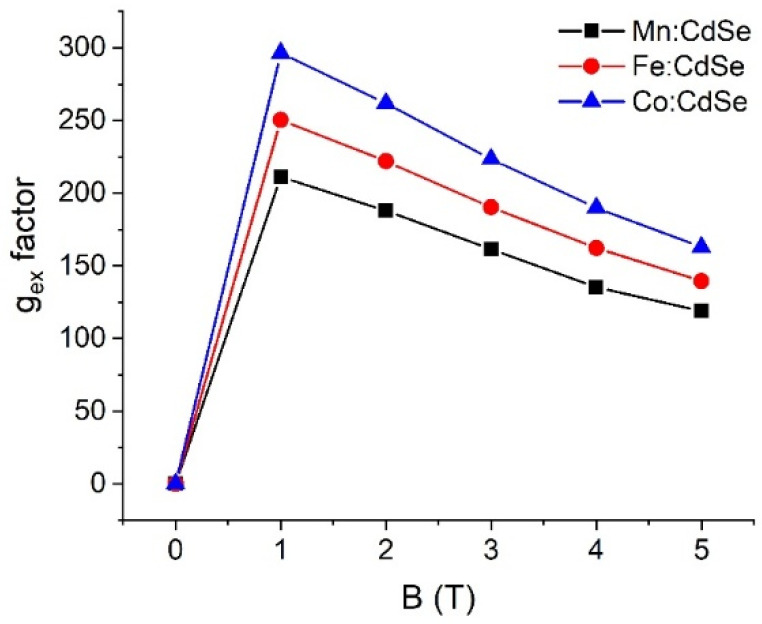
Exciton g-factor values (g_ex_) of Mn:, Fe: and Co:CdSe nanocrystals as a function of the external applied magnetic fields.

To obtain evidence of the sp-d exchange interaction induced by the species of transition metals and applied fields, it is necessary to find the g-factor values. Figure 6, Figure 7 and Figure 8 elucidate the electron, hole and exciton g-factor values of Mn:, Fe: and Co:CdSe nanocrystals under different applied magnetic fields, respectively. The electron (hole) g-factor values are described by the relation $(E_{2}-E_{1})/\mu_{B}B$, where E_1_ and E_2_ are the first and second energies of electrons (or holes), respectively. In addition, the excitonic g-factor values are calculated by the equation $(E_{\sigma^{-}}-E_{\sigma^{+}})/\mu_{B}B$. In analogy to the energies of Zeeman splitting, all g-factor values significantly improved in the presence of the applied field, and a decreasing trend was then observed with increasing applied fields. This means that electrons, holes and excitons strongly interact with the magnetic field. With increasing fields, their interactions are gradually reduced. Like the spin-splitting energies of electron states, the descending order of g_e_ values is Fe:CdSe > Mn:CdSe > Co:CdSe. Because of the trend in the energies of hole spin and Zeeman splitting, g_h_ and g_ex_ values follow the order Co:CdSe > Fe:CdSe > Mn:CdSe. Interestingly, g_h_ values are about an order of magnitude larger than g_e_ values. Therefore, the p-d exchange term contributed from holes plays an essential role in the sp-d exchange interaction over the s-d exchange term. The calculations of g-factors are considered as the qualitative guidance for the experimentalist.

**Figure 9:**
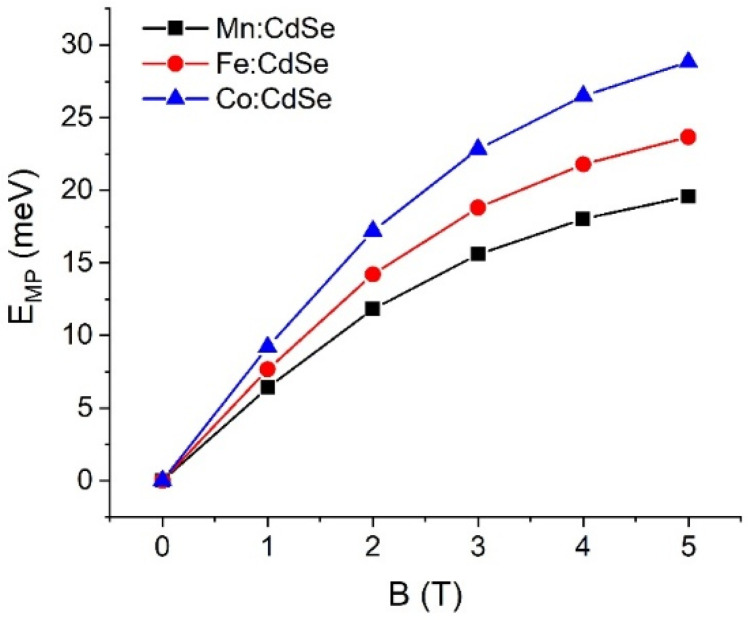
Magnetic polaron energies (E_MP_) of Mn:, Fe: and Co:CdSe nanocrystals as a function of the external applied magnetic fields.

Finally, we are particularly concerned with how the ferromagnetic orientation of the localized magnetic ion doping in the CdSe host nanocrystals is formed. To describe this phenomenon, the magnetic polaron (MP) is the direct proof to determine the ferromagnetic orientation. The energy gaining the magnetic polaron energy (E_MP_) is calculated by the equation $E_{GS}^{Non}-E_{GS}^{Mag}$, with $E_{GS}^{Non}$ and $E_{GS}^{Mag}$ being the ground-state (GS) energies of non-magnetic (Non) and magnetic (Mag) nanoparticles [56]. This description is only reliable at low temperatures. Figure 9 depicts the magnetic polaron energies of CdSe nanocrystals incorporated with Mn, Fe and Co as a function of the applied fields. With increasing magnetic fields, E_MP_ is mainly enhanced and probably reaches saturation at high fields. This means that the localized magnetic ion doping in the host nanoparticles is ferromagnetically ordered. The ferromagnetic orientation of all localized magnetic ions is tacitly assumed to be found at high applied fields. In addition, the magnetic polaron energies follow the order Co:CdSe > Fe:CdSe > Mn:CdSe. There is no comparison of our model with the experiments. We expect that the magnetic polaron energies obtained from our model and from the experiments have the same trend and magnitude order. These calculations are represented as the recommendation for the experimentalist. As a result of the calculations, the atomistic tight-binding model is used to evaluate, verify and distinguish the sp-d exchange interactions in CdSe nanocrystals incorporated with different species of transition metals. 

## 4. Conclusions

The electronic and magnetic characteristics of the CdSe nanoparticles embedded with Mn, Fe and Co under various applied magnetic fields are successfully studied by the atomistic tight-binding model with the consideration of sp-d exchange interactions. The calculations highlight that species of the transition metals and the strengths of the exterior magnetic fields are influential issues to manipulate the electronic and magnetic properties of Mn:, Fe: and Co:CdSe nanocrystals, thus facilitating their implementation into optoelectronic and spintronic devices. When external magnetic fields are applied, the spin degenerate states of both electrons and holes collapse and result in spin splitting because of the sp-d exchange term. Upon increasing the applied fields, the energies of spin splitting and Zeeman splitting are enhanced and supposed to be saturated at high fields. With growing applied magnetic fields, the single-particle gaps decrease slightly. All g-factor values are substantially enhanced in the presence of the external magnetic field and then gradually drop with increasing magnetic fields. The g_h_ values are relatively an order of magnitude larger than the g_e_ values. Therefore, the p-d exchange term contributed from holes plays a vital role in the interaction of the sp-d exchange term over the s-d exchange term from electrons. With the consideration of the reliable description, the magnetic polaron energies mainly increase and probably reach saturation at high fields. From the analysis of the magnetic polaron energies, the localized magnetic ion doping in the host nanoparticles is ferromagnetically ordered with increasing applied magnetic fields. All localized magnetic ions are assumed to display ferromagnetic alignment at the high applied fields. In terms of the transition metal species, the descending order of the electron spin-splitting energies and electron g values is Fe:CdSe > Mn:CdSe > Co:CdSe. The hole spin-splitting energies and hole g-factor values follow the order Co:CdSe > Fe:CdSe > Mn:CdSe. The order of the single-particle gaps is realized in the form of Co:CdSe > Fe:CdSe > Mn:CdSe. Caused by the energies of the electron and hole spin splitting, the Zeeman splitting energies follow in the order of Co:CdSe > Fe:CdSe > Mn:CdSe. In addition, the magnetic polaron energies follow the order Co:CdSe > Fe:CdSe > Mn:CdSe. Finally, we expect that the CdSe nanocrystals doped with various magnetic ions provide comprehensive information for readers interested in the nano-fabrication field and are prospective materials for future application in spintronic devices.

## Figures and Tables

**Table 1:** The sp-d exchange integrals for various dilute magnetic semiconductors [52].

Material	$N_{0}\alpha$ (eV)	$N_{0}\beta$ (eV)
Mn:CdSe	0.23	−1.27
Fe:CdSe	0.26	−1.53
Co:CdSe	0.28	−1.87

## Data Availability

The original contributions presented in this study are included in the article. Further inquiries can be directed to the corresponding author.
